# Functional Properties of Chitosan Conjugated with Oleic Acid and Caffeic Acid and Its Application in Oil-in-Water Emulsions

**DOI:** 10.3390/molecules31030505

**Published:** 2026-02-02

**Authors:** Tsung-Shi Yang, Tzu-Ying Ho, Tai-Ti Liu

**Affiliations:** 1 Department of Cosmeceutics, China Medical University, No. 100, Sec. 1, Jingmao Rd., Beitun Dist., Taichung 406040, Taiwan; tsyang@mail.cmu.edu.tw (T.-S.Y.); u105015019@cmu.edu.tw (T.-Y.H.); 2 Department of Food Science, Yuanpei University of Medical Technology, No. 306 Yuanpei Street, Hsinchu 30015, Taiwan

**Keywords:** chitosan, oleic acid, caffeic acid, conjugate, interfacial activity, antioxidant activity

## Abstract

The objective of this study was to develop multifunctional chitosan (CT) derivatives by conjugating oleic acid (OLA) and caffeic acid (CAF) to improve water solubility at neutral pH, enhance interfacial activity, and provide antioxidant protection in oil-in-water emulsions. Two CAF-incorporation strategies—1-(3-dimethylaminopropyl)-3-ethylcarbodiimide·HCl/N-hydroxysuccinimide (EDC/NHS)-mediated amide coupling and ascorbic acid/H_2_O_2_-initiated free radical grafting—were employed to functionalize the CT–OLA backbone. The CT–OLA–CAF conjugates generated via the free radical pathway exhibited markedly lower viscosity and interfacial tension than those produced through EDC/NHS coupling, thereby highlighting the respective advantages and limitations associated with these two synthesis approaches. Chemically, CAF incorporation substantially enhanced the antioxidant capacity of the conjugates—surpassing that of both CT and CT—OLA and conferred excellent protection to photo-oxidatively labile β-carotene in o/w emulsions.

## 1. Introduction

Chitosan is a linear polysaccharide composed of repeating units of D-glucosamine and N-acetyl-D-glucosamine, linked by β-(1→4) glycosidic bonds. Chitosan is produced by the partial deacetylation of chitin, which is primarily obtained from the waste of marine crustaceans. Chitosan has attracted considerable attention owing to its excellent biocompatibility, biodegradability, low toxicity, and versatile functionalities. Consequently, it has been employed worldwide in a broad range of applications. For instance, in the food industry, it is used as an antimicrobial agent; in edible films and coatings to control post-harvest deterioration of fruits; and as an additive, functioning as a natural flavor extender, emulsifying agent, thickener, stabilizer, or as an encapsulating material for nutraceuticals [1]. Despite these advantages, chitosan possesses several limitations, such as poor water solubility at physiological pH, low transfection efficiency, and insufficient functionalities for some intended applications [2]. Therefore, various chemical modifications have been employed to overcome its limitations and enhance its functionality.

Regarding the use of chitosan in emulsions, it can stabilize them through its viscosity and emulsifying ability. However, its high Hydrophilic–Lipophilic Balance (HLB) value of 34–36.7 indicates that its molecular structure is more hydrophilic and, therefore, not a suitable emulsifier for forming stable O/W emulsions, which typically require an HLB value of around 10–12 according to empirical observations [3]. To enhance the emulsifying ability of chitosan, negatively charged emulsifiers were combined with the positive groups of chitosan through electrostatic interaction to form multilayered emulsions [4,5]. Another approach involves using chitosan in the form of particles between the oil and water phases to stabilize emulsions, called Pickering emulsions. Caffeic acid has been grafted onto chitosan using free radical mediated-method to provide antioxidant activity and is used in the o/w Pickering emulsion. A quick appearance of creaming phase separation was observed on the first day, which is considered a characteristic property of Pickering emulsion [6]. The stability of particle-stabilized Pickering emulsions is notably governed by intrinsic factors—including particle wettability, morphology, size, zeta potential, concentration, and packing configuration—as well as extrinsic conditions such as pH, additives, and other environmental variables [7]. Therefore, selecting appropriate materials and mixing conditions for preparing such emulsions becomes more complex.

Lipid oxidation is an interfacial process that begins at the interface between oil and water. At this boundary, unsaturated fatty acids from the oil phase interact with prooxidants, such as trace metal ions, present in the aqueous phase. The emulsification process creates a large interfacial area between the oil and aqueous phases, which facilitates more interactions between the reactants, potentially accelerating the rate of lipid oxidation [8]. If an antioxidant is located at the interface, its efficiency in protecting against lipid oxidation can be enhanced. Therefore, an emulsifier with antioxidant activity would be an ideal choice for such emulsions. Chitosan has limited antioxidant activity; thus, various phenolic compounds have been used to modify it and enhance this activity. For instance, Chitosan was grafted with gallic acid, caffeic acid, gentisic acid, and sinapic acid using the 1-ethyl-3-(3′-dimethylaminopropyl) carbodiimide/hydroxybenzotriazole (EDC/HOBt)-mediated method to enhance its antioxidant and antimicrobial activities, with the degree of substitution varying depending on the type of compound [9]. These conjugated compounds were not used in emulsions for any application. Similarly, chitosan was grafted with caffeic acid and 3,4-dihydroxybenzoic acid using EDC·HCl/HOBt as the coupling agent. The phenolic acid-grafted chitosans exhibited improved emulsification properties as well as enhanced antioxidant and antimicrobial activities relative to native chitosan [10]. Using a different reagent, the conjugate of chitosan and caffeic acid was prepared via caffeic acid chloride, with dimethylaminopyridine as the catalyst. The caffeic acid-grafted chitosan exhibited higher antioxidant activity than chitosan, but the creaming index of this conjugate, when used to stabilize the O/W emulsion, was higher than that of chitosan [11]. The reported properties of the chitosan–caffeic acid conjugate appear to be inconsistent with respect to its solubility. In one study, it was used as particulate stabilizers in o/w emulsions, whereas in the other, it was water-soluble and functioned as a conventional emulsifier. This discrepancy may arise from differences in the synthesis methods of the conjugate. Therefore, in this work, the properties of chitosan conjugated with caffeic acid are re-examined in the presence of fatty acids incorporated into the chitosan structure.

Fatty acids have a hydrocarbon chain structure and a carboxyl group, which provide hydrophobicity and serve as reactive sites for synthesis with other molecules to form derivatives such as surfactants, including emulsifiers. Chitosan has been modified with various fatty acids to enhance its properties: grafted with linoleic acid to form self-aggregated particles for improved trypsin adsorption [12], with linolenic acid to increase protein loading capacity [13], and with stearic acid to achieve stable Pickering emulsions [14]. Comparatively, there has been limited research combining chitosan, fatty acids, and phenolic compounds into a single molecule for use as an emulsifier in o/w emulsions. Chitosan grafted with stearic acid and gallic acid has been studied in o/w emulsions for protecting labile compounds from oxidation [15]. This research aims to contribute further insights into this area. Oleic acid has potential health benefits, such as the maintenance of normal blood cholesterol levels, anti-inflammatory effects, and improved insulin sensitivity [16]. In addition to its health benefits, oleic acid has better liquidity than stearic acid at room temperature due to its unsaturated structure. Moreover, it exhibits better oxidative stability than both linoleic acid and linolenic acid. Consequently, oleic acid was selected for use in this study.

To achieve these goals, the study focused on modifying chitosan by grafting oleic acid and caffeic acid to improve its solubility, emulsion stability, and antioxidant activity. Additionally, two distinct approaches—free radical and EDC/NHS grafting methods—were employed to synthesize the chitosan–caffeic acid conjugate, with oleic acid initially incorporated into the chitosan molecules, and to compare their effects on the properties of the conjugate.

## 2. Results and Discussion

### 2.1. Synthesis of CT–OLA

The synthetic route of CT–OLA begins with the activation of the carboxyl group of OLA by EDC, forming an unstable O-acylisourea intermediate. Upon the addition of NHS, this intermediate is converted into a more stable NHS ester [17], which subsequently reacts with the amine groups of CT to yield the final CT–OLA conjugate. Theoretically, the molar ratio of EDC to NHS in the reaction is 1:1, and this ratio was applied in this study. However, the ratios of CT and OLA to EDC/NHS were varied. The stoichiometric ratios of CT, OLA, and EDC/NHS are summarized in Table 1. The grafting efficiency of CT–OLA was assessed by determining the degree of amino substitution (%), based on the reaction of CT amino groups with TNBS.

Regarding the synthesis of CT–OLA, with the amount of CT fixed, as the ratio of OLA to EDC/NHS was less than or equal to 1, the synthesized products of the reaction exhibited some precipitation when the reacted mixture was dialyzed in the water. The degree of precipitation was recorded using a ‘+’ sign, with a higher number indicating more severe precipitation. The ratios among the reactants must be balanced to achieve successful synthesis of the conjugates. It has been reported that O-acylisourea can react not only with primary amines and NHS, but also with EDC and water [18]. The hydrolysis of the intermediate in water decreases the efficacy of EDC. Furthermore, excess EDC can cause O-acylisourea to convert into N-acylurea, thereby decreasing the amount of activated acyl groups available for subsequent reactions. In this experiment, the conditions used for CT-OLA-5, with a slightly higher ratio of OLA to EDC/NHS, yielded satisfactory results. The reason may be that OLA forms an amide bond with CT, which reduces intermolecular interactions and consequently decreases crystallinity, making the material more easily dispersed. Additionally, OLA introduces hydrophobicity and enhances surface activity, as evidenced by its ability to lower surface tension (Table 1). The increased surface activity may promote micelle formation by the conjugated molecule, thereby improving its dispersion in neutral aqueous solution. To further increase the amino substitution of chitosan (CT) with OLA, the primary conjugate CT–OLA–5 was subjected to a second conjugation with OLA to obtain CT–OLA–6. Subsequently, CT–OLA–6 underwent a similar reaction to yield the CT–OLA–7 conjugate. The results in Table 1 show that the amino substitution increased by approximately 55% from CT–OLA–5 to CT–OLA–6, and by 40% from CT–OLA–6 to CT–OLA–7. In contrast, the reaction yield decreased by about 3.8% and 3.2%, respectively. This indicates that when the already conjugated CT–OLA was subjected to further reaction with OLA, the grafting efficiency was enhanced compared to a single-stage reaction. With more OLA conjugated to CT, the viscosity markedly increased while the surface tension decreased, which is favorable for the conjugate to be used in emulsion systems. Considering the degree of amino substitution, yield loss, and the availability of amino groups for the subsequent synthesis of CAF, CT–OLA–5 was adopted for the synthesis of CT–OLA–CAF.

### 2.2. Synthesis of CT–OLA–CAF

The synthetic route of CT–OLA–CAF via the EDC/NHS coupling method followed the same procedure as that used for CT–OLA synthesis, with the exception that the grafting efficiency of CAF was subsequently quantified through total phenolic content analysis of the resulting conjugate. In this reaction, the amount of CT–OLA was kept constant, while the ratios of CAF to EDC/NHS were adjusted as listed in Table 2. The results indicate that when the molar ratio of CAF to EDC/NHS was less than or equal to 1, the grafting efficiency of CT–OLA–CAF synthesis decreased. This behavior is consistent with that observed in the synthesis of CT–OLA, indicating that the amount of the grafting reactant must be greater than that of EDC/NHS to achieve a satisfactory synthesis result.

With respect to the synthesis conditions of CT–OLA–CAF via the free-radical grafting method, the only parameter altered was the concentration of H_2_O_2_. The reaction mechanism involves the formation of ascorbyl radicals [19], which subsequently attack the CT molecules to generate radical sites. These CT radicals can then be quenched by the antioxidant CAF, leading to its covalent attachment to the polymer. Therefore, the concentration of ascorbyl radicals plays a crucial role in determining the efficiency of the grafting reaction. When the H_2_O_2_ concentration was doubled, the grafting ratio decreased. This reduction may be due to excess hydrogen peroxide altering the redox equilibrium of the system, possibly accelerating the consumption of ascorbyl radicals into dehydroascorbic acid [20] and consequently lowering the effective radical concentration required for grafting.

### 2.3. NMR Spectra of CT, CT–OLA, and CT–OLA–CAF

**Figure 1:**
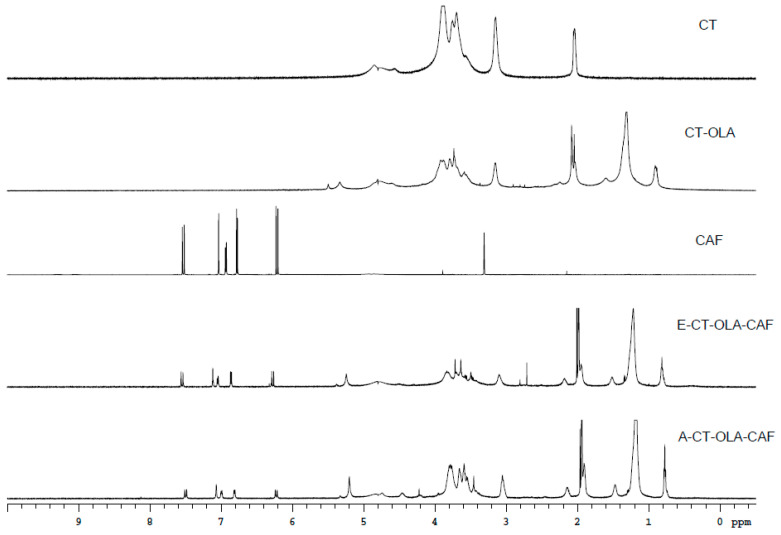
NMR spectra of CT, CAF, and CT-conjugates.

When the NMR spectra of CT, CT–OLA, E-CT–OLA–CAF, and A-CT–OLA–CAF are compared, as shown in Figure 1, distinct new signals appear in the CT–OLA spectrum relative to CT. Peaks in the range of δ 0.8–1.6 ppm correspond to the terminal methyl and methylene groups of the aliphatic chain, confirming the successful conjugation of OLA onto the CT backbone. In addition, both CAF–modified samples (E-CT–OLA–CAF and A-CT–OLA–CAF) exhibit aromatic proton resonances between δ 6.5–7.5 ppm and vinyl proton signals at δ 6.0–6.3 ppm, characteristic of CAF structures. These spectral features collectively verify the incorporation of CAF, demonstrating that both modification strategies effectively graft OLA and CAF onto CT.

### 2.4. Physical Properties of CT–OLA–CAF

The physical properties of CT–OLA–CAF synthesized by two different methods, together with those of CT and CT–OLA, are summarized and compared in Table 2. E-CT–OLA–CAF–1, which exhibited the highest grafting ratio among the conjugates synthesized via the EDC/NHS coupling method, showed a slight but statistically insignificant increase in viscosity compared with CT–OLA. This observation suggests that the attachment of CAF onto the conjugate does not markedly influence its rheological behavior, indicating that the viscosity is predominantly governed by the hydrophobic oleic acid moieties rather than the phenolic CAF groups. OLA possesses a long hydrophobic alkyl chain (C_18_). Upon grafting onto the hydrophilic chitosan backbone, intermolecular hydrophobic associations are likely to occur in the aqueous medium. These hydrophobic domains tend to interact with each other, resulting in transient physical cross-linking or aggregation among the polymer chains, thereby enhancing the internal friction within the system and consequently increasing its viscosity. In contrast, A-CT–OLA–CAF, prepared through the free-radical grafting method, presented a pronounced decrease in viscosity relative to both CT and CT–OLA. On the other hand, this decrease is likely attributed to partial degradation or depolymerization of chitosan chains during the radical process [21], which reduces molecular entanglement and weakens intermolecular interactions, consequently lowering the viscosity of the resulting conjugate.

With respect to surface tension, compared with CT, the surface tension decreased after conjugation with OLA, owing to the increased hydrophobicity. Furthermore, upon incorporation of CAF, both E-CT–OLA–CAF and A-CT–OLA–CAF exhibited lower values than CT–OLA, suggesting that the addition of CAF to the CT–OLA molecular structure further enhances its surface activity. This improvement may be attributed to the introduction of hydrophobic CAF moieties, which promote more efficient orientation at the air–water interface and reduce intermolecular cohesion, thereby lowering the surface tension [22]. The decrease in viscosity facilitates molecular diffusion and rearrangement at the air–water interface. When the viscosity is lower, the intermolecular friction within the solution is reduced, allowing the surface-active molecules to migrate more rapidly toward the interface. As a result, the adsorption equilibrium can be reached faster, leading to a greater surface coverage. Moreover, enhanced molecular mobility enables the grafted chains to reorient more efficiently at the interface, forming a compact and energetically favorable alignment that minimizes interfacial free energy. Consequently, a lower viscosity contributes to faster adsorption kinetics and more effective surface packing, thereby resulting in a decrease in surface tension [23].

Regarding the zeta potential, both CT conjugated with OLA and CT–OLA further conjugated with CAF via the EDC/NHS method exhibited similar zeta potential values, which were higher than that of native CT. This increase might result from the hydrophobic segment of OLA inducing molecular aggregation, thereby resulting in a higher local density of positive charges at the colloidal interface. It has been reported that hydrophobic interactions can bring chitosan–fatty acid conjugate molecules closer together, thereby increasing the local charge density. If this effect outweighs the reduction in amino groups caused by substitution, the overall positive charge will increase; otherwise, it will decrease [24]. In this study, the results suggest that the former effect predominated. Comparatively, the zeta potential of A-CT-OLA-CAF was lower than that of E-CT-OLA-CAF. This is probably because the molecular structure of CT was partially degraded during the synthesis of A-CT-OLA-CAF by free radicals, resulting in a reduction in molecular weight, as also evidenced by the decrease in viscosity of A-CT-OLA-CAF. Previous studies have reported an inverse relationship between the molecular weight of chitosan and its zeta potential [25].

### 2.5. Antioxidant Activity of CT Conjugates

#### 2.5.1. DPPH Radical Scavenging Activity

DPPH is a stable free radical that is widely used to evaluate the antioxidant capacity of compounds because the assay is simple, rapid, reproducible, and does not require sophisticated instrumentation. When an antioxidant donates a hydrogen atom or an electron to the DPPH radical, the DPPH molecule is reduced to the corresponding hydrazine (DPPH-H), leading to a color change from deep violet to pale yellow. CT and CT–OLA did not exhibit noticeable scavenging activity within the tested concentration range, whereas CAF showed a pronounced effect. Therefore, it can be inferred that the antioxidant activity of CT–OLA–CAF primarily originates from the incorporated CAF moieties.

Although the CAF content in CT–OLA–CAF increased by 94.1% from E-CT–OLA–CAF-3 to E-CT–OLA–CAF-1, the DPPH radical scavenging activity exhibited only a 42.7% increase (Table 3). This disproportionate improvement can be attributed to structural rearrangements resulting from the increased hydrophobicity of the conjugate imparted by CAF. Based on the comparison of their surface tension values, the reduction in surface tension likely facilitated molecular self-assembly or inward folding of the hydrophobic domains, leading to the partial burial of CAF moieties within the hydrophobic core of the conjugate. Consequently, the effective exposure of CAF to DPPH radicals in methanol was reduced, resulting in a less pronounced increase in scavenging activity despite the higher CAF loading. The grafting ratio of A-CT-OLA-CAF-1 (6.89%) was slightly higher than that of E-CT-OLA-CAF-2 (6.84%), yet its DPPH radical scavenging activity was considerably greater. This difference may be due to some CAF molecules being grafted onto the hydroxyl (–OH) groups of chitosan, away from the influence of conjugated OLA, which allowed better accessibility to DPPH radicals in the polar methanol medium. This effect appears to outweigh the impact of hydrophobicity, even though A-CT-OLA-CAF-1 exhibited a relatively lower surface tension.

#### 2.5.2. Hydrogen Peroxide Scavenging Activity

Hydrogen peroxide (H_2_O_2_) is an important reactive oxygen species (ROS) produced endogenously during normal cellular metabolism. While H_2_O_2_ acts as a redox signaling molecule at low concentrations to regulate various cellular processes, its excessive accumulation induces oxidative stress. Moreover, H_2_O_2_ can generate highly reactive hydroxyl radicals (•OH) through Fenton-type reactions in the presence of transition metals. These radicals can cause severe oxidative damage to vital cellular macromolecules such as lipids, proteins, and polysaccharides. Therefore, the antioxidant activity of CT–OLA–CAF was evaluated to assess its protective potential against H_2_O_2_-induced oxidative stress. The CAF content in CT–OLA–CAF increased by 94.1% from E-CT–OLA–CAF-3 to E-CT–OLA–CAF-1; however, the H_2_O_2_ scavenging activity increased by only 24.68% (Table 3). The efficiency of H_2_O_2_ scavenging was notably lower than that observed for DPPH radical scavenging. This discrepancy may be attributed to the distinct reaction mechanisms involved: DPPH radicals readily accept hydrogen atoms or electrons from phenolic hydroxyl groups, whereas H_2_O_2_, a non-radical ROS with lower reactivity, is primarily reduced through electron transfer processes. Consequently, the antioxidant performance depends more on the redox potential of the compound and the accessibility of its reactive sites rather than solely on its total phenolic content. This interpretation is further supported by the observation that the reducing ability increased by 64.05% based on ascorbic acid equivalence and by 61.65% based on caffeic acid equivalence (Table 3), both of which are lower than the increase in grafting ratio.

### 2.6. Stability of Emulsions Stabilized by CT and Its Conjugates

To evaluate the emulsifying performance and stability of CT and its conjugates, oil-in-water (O/W) emulsions stabilized by these materials were subjected to accelerated stability testing. The oil-droplet size and distribution were analyzed to assess the effect of molecular modification on emulsion stability. The results in Table 4 show that the emulsion stabilized by unmodified CT exhibited the largest droplet size and the broadest size distribution among the CT samples and their conjugates. In contrast, grafting CT with OLA markedly reduced the droplet size by approximately 45% and decreased the PDI value by 43% (Table 4). Furthermore, covalent conjugation of CAF to CT–OLA did not cause significant changes in droplet size or size distribution. This phenomenon was consistent with the minimal change in surface tension observed for CT–OLA conjugates after CAF grafting via the EDC/NHS coupling method. These results demonstrate that OLA plays the dominant role in imparting hydrophobicity, which primarily reduces surface activity and consequently leads to smaller droplet size and narrower size distribution. In contrast, the CT–OLA–CAF conjugate synthesized via the free-radical grafting method showed markedly smaller droplet sizes and narrower distributions, with reductions of 54.4% in mean size and 57.1% in PDI, respectively. The reason might be ascribed to the much lower surface tension of A-CT–OLA–CAF compared with that of E-CT–OLA–CAF. In addition, the lower molecular weight of A-CT–OLA–CAF may also facilitate the mobility of the molecules toward the O/W interface, leading to the formation of smaller droplets.

The effect of heating on the oil-droplet size and distribution of CT and its conjugate-stabilized emulsions is summarized in Table 4. After heat treatment, the droplet sizes of CT-, CT–OLA-, E-CT–OLA–CAF-, and A-CT–OLA–CAF-stabilized emulsions increased by 5.92%, 5.28%, 3.01%, and 15.75%, respectively. The relatively larger increase observed for the A-CT–OLA–CAF system may be attributed to its lower viscosity among the emulsions, which could reduce the resistance to droplet coalescence or deformation during heating. Meanwhile, the PDI values of all samples showed no significant changes after heat treatment, indicating that the overall droplet size distribution remained stable. Regarding the effect of centrifugation, the droplet size of the CT-stabilized emulsion increased by 3.62%, whereas those of all CT conjugate-stabilized emulsions changed by less than 0.3%, with no significant variations observed in their PDI values. The present findings demonstrate that the modification of CT with OLA and CAF significantly improves its performance in oil-in-water emulsions, exhibiting enhanced stability even under accelerated storage conditions.

**Figure 2:**
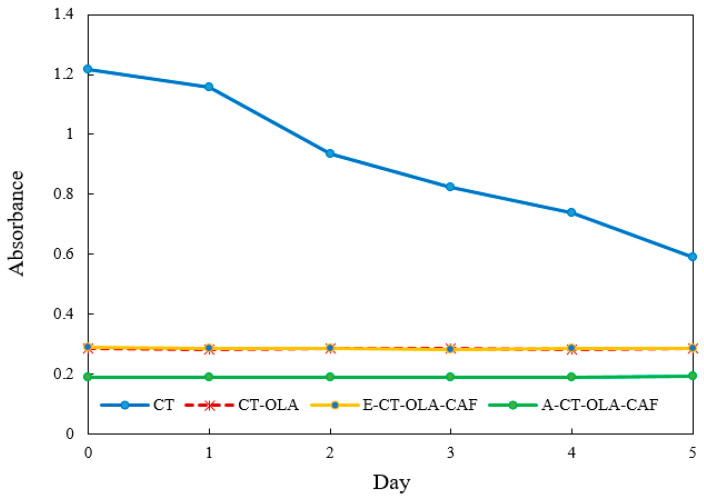
Effect of ionic strength (400 mM NaCl) on the stability of emulsions stabilized by CT and CT Conjugates.

The stability of emulsions is highly sensitive to the ionic environment, particularly in systems stabilized by polymeric emulsifiers bearing surface charges. Ionic strength can influence electrostatic interactions at the oil–water interface, modulate the thickness and integrity of the interfacial film, and alter the zeta potential of dispersed droplets, which in turn affects their ability to resist coalescence and maintain dispersion stability. Figure 2 illustrates the influence of increasing ionic concentrations on the stability of emulsions stabilized by CT and its conjugates during storage. Emulsion stability was evaluated by changes in turbidity, measured as absorbance, where a lower absorbance value indicates reduced stability. As ionic strength increased, noticeable variations in emulsion stability were observed for the CT-based system, which showed pronounced sensitivity to ionic stress with a 48.5% decrease in absorbance after five days. In contrast, conjugates modified with OLA and CAF exhibited enhanced resistance, maintaining structural stability under high-salt conditions. The superior stability of CT–OLA and E-CT-CAF conjugates under ionic stress may also be associated with their higher zeta potential values. The increased surface charge enhances electrostatic repulsion among droplets, counteracting the screening effect of added ions. Even when the electrical double layer is partially compressed at high ionic strength, the initially high zeta potential of CT–OLA derivatives helps maintain sufficient repulsive energy to prevent droplet coalescence. Although A-CT–OLA–CAF exhibited a zeta potential similar to that of unmodified CT, its emulsion stability was comparable to CT–OLA under ionic stress. This phenomenon can be attributed to its significantly lower surface tension, which enhances the adsorption of polymer molecules at the oil–water interface and facilitates the formation of a more compact and cohesive interfacial film. The reduced surface tension lowers the interfacial energy barrier, promoting the spontaneous spreading of the emulsifier on the droplet surface and thereby preventing coalescence. In addition, the presence of hydrophobic OLA chains and phenolic CAF moieties reinforces interfacial packing through hydrophobic and hydrogen-bonding interactions. Consequently, despite having a relatively lower zeta potential, A-CT–OLA–CAF achieves strong steric stabilization and maintains excellent emulsion stability.

### 2.7. Photostability of β-Carotene in CT and CT Conjugate Emulsions

**Figure 3:**
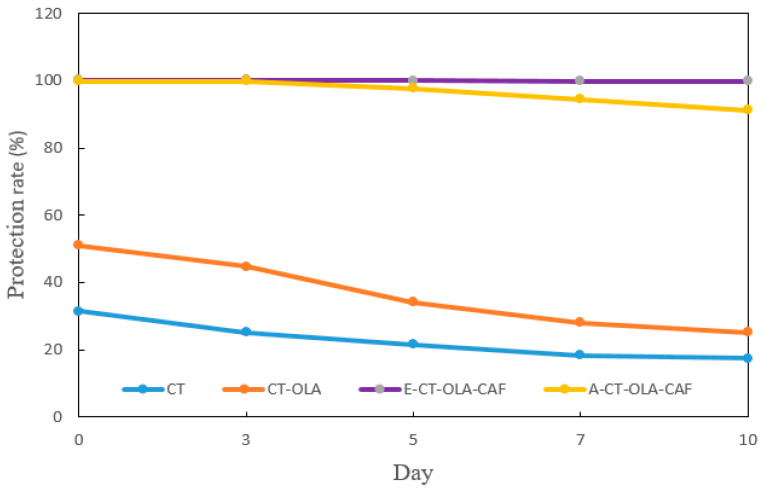
Photostability of β-carotene in CT- and CT-Conjugate-stabilized emulsions during storage under illumination.

β-Carotene is important in food not only as a nutrient (provitamin A) but also as a natural antioxidant and colorant, contributing to both health benefits and product quality. However, its highly conjugated polyene structure makes it particularly sensitive to oxidative degradation, especially photooxidation under light exposure, which leads to rapid loss of color and antioxidant activity. Given this susceptibility, the photostability of β-carotene in CT and CT-conjugate emulsions was evaluated to assess their protective effects. The protection rate of β-carotene in the CT emulsion decreased from approximately 31% to 17% after 10 days of storage, whereas that in the CT-OLA emulsion decreased from approximately 51% to 25% (Figure 3). These results indicate that incorporating OLA improves the photostability of β-carotene, particularly during the early storage period. This enhancement is likely due to the increased hydrophobicity and improved interfacial activity provided by OLA, which facilitates a deeper partitioning of β-carotene into the oil droplet core. As a result, β-carotene experiences reduced light exposure and thus undergoes less photo-degradation. Furthermore, the incorporation of CAF markedly improved photoprotection; the β-carotene content in the E-CT–OLA–CAF emulsion showed no significant degradation, and the A-CT–OLA–CAF emulsion likewise retained over 90% of β-carotene after 10 days of storage. Compared with CT–OLA, the pronounced enhancement in the photostability of β-carotene observed after the incorporation of CAF into the CT–OLA structure can be attributed to the intrinsic photoprotective properties of CAF. CAF possesses a strong ultraviolet-absorbing chromophore, with a reported maximum absorption band centered around 325 nm [26], which lies within the UVB region responsible for initiating β-carotene photo-oxidation. Upon conjugation, CAF becomes covalently anchored to the CT-OLA backbone, allowing it to localize at or near the oil–water interface of the emulsion droplets. This interfacial positioning enables CAF to function as an effective UV screen, attenuating photon flux before it reaches the β-carotene molecules within the oil phase. Beyond direct UV absorption, CAF also contributes to photoprotection through secondary radical-scavenging pathways. During photolysis, β-carotene readily undergoes oxidative degradation initiated by ROS. The phenolic structure of CAF, characterized by its o-diphenolic (catechol) moieties, can effectively quench ROS and intercept lipid-derived radicals generated at the oil–water interface. This dual mechanism—photon attenuation coupled with radical scavenging—synergistically reduces the propagation rate of photo-oxidative reactions. Collectively, the combination of interfacial localization, strong UVB absorption capacity, and the redox-active catechol functionality results in substantially enhanced protection of β-carotene in the CT–OLA–CAF conjugate emulsions.

## 3. Materials and Methods

### 3.1. Materials

Chitosan (80% deacetylated; MW 50–190 kDa), L-ascorbic acid, 1,1-diphenyl-2-picrylhydrazyl, oleic acid, β-carotene, Folin–Ciocalteu reagent, sodium carbonate, and 2,4,6-trinitrobenzenesulfonic acid (TNBS) were purchased from Sigma-Aldrich Co. (St. Louis, MO, USA). 1-(3-Dimethylaminopropyl)-3-ethylcarbodiimide·HCl was obtained from Matrix Scientific (Columbia, SC, USA). N-hydroxysuccinimide was obtained from Alfa Aesar Co. (Ward Hill, MA, USA). Caffeic acid was purchased from Acros Organics (Geel, Belgium). Potassium bromide and acetic acid were obtained from Nihon Shiyaku Industries Ltd. (Kyoto, Japan). Hydrogen peroxide was purchased from Honeywell International Inc. (Morristown, NJ, USA). Potassium ferricyanide and sodium bicarbonate were obtained from J.T. Baker (Center Valley, PA, USA).

### 3.2. Synthesis of Chitosan-Oleic Acid (CT-OLA) and Chitosan-Oleic Acid-Caffeic Acid (CT-OLA-CAF) Conjugates via the EDC/NHS-Mediated Coupling

Initially, 1 g of chitosan was dissolved in 40 mL of 1% (*v*/*v*) acetic acid solution. Subsequently, 100 mL of methanol was added to the chitosan solution and mixed thoroughly. In a separate container, OLA, EDC, and NHS were dissolved in 30 mL of methanol at predetermined molar ratios and stirred at 60 °C for 1 h. The OLA/EDC/NHS solution was then slowly added dropwise into the chitosan solution while maintaining the reaction temperature at 60 °C with stirring at 400 rpm. The grafting reaction was allowed to proceed for 6 h under these conditions [27]. Upon completion, the reaction mixture was purified by dialysis. Specifically, it was transferred into a cellulose dialysis membrane (MWCO 12–14 kDa; Biomate^TM^, Taipei, Taiwan) and dialyzed against deionized water at 4 °C for 4 d. The dialysis water (4 L) was replaced twice daily to ensure efficient removal of unreacted reagents and by-products. After dialysis, the purified CT-OLA product was collected, and the samples were pre-frozen at −20 °C for 1 d, followed by freeze-drying at −80 °C for 3 d. The synthesis of CT-OLA-CAF was carried out using CT-OLA and CAF as reactants, following a procedure similar to that described above.

### 3.3. Amino Substitution of Chitosan

A 1 mg/mL sample was first prepared in 1% acetic acid. In a 3 mL vial, 200 μL of the sample solution was added, followed by 400 μL of 4% NaHCO_3_ aqueous solution and 400 μL of 0.1% TNBS solution. The mixture was vortexed to ensure uniform mixing and then incubated at 40 °C for 90 min. After the reaction, the sample was protected from light and cooled to room temperature for 10 min. The reaction was then terminated by adding 120 μL of 1 N HCl. Subsequently, 200 μL of the resulting solution was transferred to a 96-well plate, and the absorbance at 344 nm was measured using a microplate spectrophotometer (Hidex Sense, Turku, Finland) [24].

Amino substitution was calculated as follows:

Amino substitution (%) = [(A_T_ − Ac)/A_T_] × 100,

where A_T_ is the absorbance of CT and Ac is the absorbance of CT-OLA.

### 3.4. Synthesis of CT-OLA-CAF Conjugates via the Free Radical Grafting Method

The synthesis of CT-OLA-CAF was adapted from reference [6] with modifications. CT-OLA (0.5 g) was dissolved in 50 mL of 1% (*v*/*v*) acetic acid and stirred at 400 rpm for 30 min. Ascorbic acid (0.054 g) was then added, followed by 1 mL of H_2_O_2_. Separately, CAF (0.25 g) was dissolved in 5 mL of methanol and subsequently introduced into the reaction mixture, which was allowed to proceed in the dark for 24 h. The crude product was purified by dialysis against deionized water for 4 d, with water replaced twice daily, and then lyophilized to obtain the CT-OLA-CAF conjugates.

### 3.5. Determination of Grafting Ratio of CT-OLA-CAF

CT-OLA-CAF was dissolved in deionized water to obtain a neutral aqueous solution (2 mg/mL), and 120 μL was mixed with 240 μL of 1 N Folin–Ciocalteu reagent. After incubation in the dark for 5 min, 480 μL of 12.4% Na_2_CO_3_ solution was added, and the mixture was further incubated in the dark for 30 min. The reaction mixture was then centrifuged at 4000× *g* for 3 min, and 200 μL of the supernatant was transferred to a 96-well plate for absorbance measurement at 760 nm [28]. A calibration curve was constructed using CAF standards under identical conditions, and the CAF content of CT-OLA-CAF was calculated to determine its grafting ratio.

### 3.6. NMR Analysis

CT-OLA-CAF was dissolved in D_2_O; OLA in CDCl_3_; and CT as well as CT-OLA in D_2_O containing 1% CD_3_COOD. The ^1^H-NMR spectra of these samples were acquired using an NMR spectrometer (Agilent DD2, 600 MHz, Agilent Technologies, Inc., Santa Clara, CA, USA).

### 3.7. Viscosity Measurement

The sample was dissolved in a 1% acetic acid solution (1.5 mg/mL), and its viscosity was measured using a viscometer (RVDV-II CP, Brookfield Engineering Laboratories, Inc., Middleboro, MA, USA) at 25 °C. The rotor specification was CPE-42, with a gap setting of 10 mm and a rotational speed of 200 rpm. A volume of 1 mL of the sample solution was drawn and placed in the sample chamber for viscosity measurement. The viscosity value was then recorded.

### 3.8. Measurement of Surface Tension

An aliquot (10 mL, 1 mg/mL) of the sample solution was placed in a circular glass dish (ID: 5 cm) mounted on a height-adjustable stage. Surface tension was measured using the Du Noüy ring method with a platinum ring submerged beneath the liquid surface. The stage was gradually lowered until the meniscus detached from the ring, and the surface tension was automatically recorded with a tensiometer (KSV SIGMA 703D, Biolin Scientific, Stockholm, Sweden).

### 3.9. Analysis of Zeta Potential

The samples (0.2 mg/mL) were prepared in 1% acetic acid. Zeta potential was determined using a Zetasizer Nano ZS90 (Malvern Instruments Ltd., Malvern, UK). Measurements were based on electrophoretic mobility, calculated with the Henry equation assuming F(κa) = 1.5. The refractive index and viscosity of the medium at 25 °C were 1.330 and 0.8872 cP, respectively.

### 3.10. DPPH Analysis

The samples were prepared at various concentrations. A 40 μL aliquot of each sample was transferred to a 1.5 mL centrifuge tube, followed by the addition of 360 μL of 0.4 mM DPPH methanol solution. The reaction was allowed to proceed for 30 min in the dark. Afterward, 200 μL of the reaction mixture was transferred to a 96-well plate, and the absorbance was measured using a microplate reader set to a scanning wavelength of 517 nm. The DPPH scavenging activity was calculated using the following formula:

DPPH Scavenging Activity (%) = [(Ac − As)/Ac] × 100,

where Ac is the absorbance of the control, As is the absorbance of the sample group. The concentration required to inhibit 50% of oxidation (IC_50_) was determined from the regression curve of the sample’s scavenging activity.

### 3.11. Analysis of Hydrogen Peroxide Scavenging Activity

This assay was conducted with reference to the method described in [29], with modifications. Sample solutions of CT and its conjugates were prepared at various concentrations. In addition, 20 mM H_2_O_2_ solution in pH 7.4 phosphate-buffered saline (PBS) were prepared. An aliquot of 100 μL of the sample solution was mixed with 100 μL of 20 mM H_2_O_2_ in a 96-well UV microplate and incubated in the dark for 10 min. The absorbance was then measured at 230 nm using a microplate reader. Scavenging activity was assessed using the following formula:

Scavenging activity (%) = [(Ac − As)/Ac] × 100,

where Ac is the absorbance of the control and As is the absorbance of the sample.

### 3.12. Ferric Ion Reducing Power Measurement

This study referenced and modified the experimental method reported in [30]. A 1 mg/mL sample solution was prepared, along with 1% potassium ferrocyanide solution, 10% trichloroacetic acid (TCA) solution, 0.2 M pH 6.6 PBS, and 0.1% FeCl_3_ solution. A 100 μL aliquot of the sample solution was placed into a 1.5 mL centrifuge tube, followed by the addition of 100 μL of 0.2 M pH 6.6 PBS and 100 μL of 1% TCA. The mixture was then incubated in the dark at 50 °C for 20 min. After the reaction, the sample was cooled in the dark for 10 min, and then 100 μL of 10% TCA was added. The mixture was centrifuged at 5000× *g* for 5 min. A new 1.5 mL centrifuge tube was prepared, to which 100 μL of the supernatant, 100 μL of distilled water, and 0.1% FeCl_3_ were added and reacted for 10 min. Finally, 200 μL of the reaction solution was transferred to a 96-well plate, and absorbance was measured at 700 nm. Ascorbic acid and CAF were used as standards for comparison. A greater absorbance indicates a higher reducing power.

### 3.13. Emulsion Stability Test

The sample was dissolved in a 1% acetic acid solution (0.5 wt%). Soybean oil (2 wt%) was added to the aqueous phase and homogenized in an ice bath using a high-speed homogenizer at 19,000 rpm for 10 min. The resulting emulsions were transferred into glass vials, sealed with aluminum caps containing septa, and left to stand for 24 h to eliminate foam and allow stabilization. Subsequently, the vials were inverted, and aliquots were withdrawn by inserting a syringe through the septum at the bottom of each vial. The needle was positioned at a fixed mark on the vial to ensure consistent sampling depth, while the vial remained stationary and undisturbed throughout the process. Emulsion stability was assessed by monitoring turbidity changes, with absorbance at 600 nm measured using a spectrophotometer.

### 3.14. Emulsion Stability Against Ionic Strength

The stability of the emulsion against ionic strength was evaluated by mixing it with a 400 mM NaCl aqueous solution [31]. The original emulsion was diluted with water to an oil content of 0.2% and subsequently combined with the NaCl solution at a volume ratio of 1:5. The resulting emulsions were transferred into glass vials and subsequently subjected to the procedures described in the emulsion stability test above. The absorbance at 600 nm was recorded on days 0, 1, 2, 3, 4, and 5. Variations in absorbance were used to assess the effect of ions on emulsion stability.

### 3.15. Effect of Centrifugation on Emulsion Stability

The effect of centrifugation on emulsion stability was evaluated using 15 mL centrifuge tubes containing 8 mL of emulsion. Samples were centrifuged at 7750× *g* for 20 min. After centrifugation, the emulsions were transferred into glass vials (1.5 cm in diameter), capped, and inverted for 20 h. The emulsions were subsequently sampled at a fixed mark on the vials, and the particle size was determined to evaluate the effect of centrifugation on emulsion stability.

### 3.16. Evaluation of the Thermal Stability of the Emulsion

The thermal stability of the emulsion was evaluated by heating 8 mL of emulsion in glass vials at 80 °C for 30 min. After heating, the vials were inverted and left to stand for 20 h. The particle size of the emulsions was determined according to the procedures described above.

### 3.17. Preparation of β-Carotene Emulsion and Photostability Test

The method was adapted from [32] with modifications. CT and its conjugates (0.5 wt%) in 1% acetic acid were used to prepare the aqueous phase. β-Carotene (0.125 wt%) was dissolved in medium-chain triglycerides and used as the oil phase. The oil phase (2 wt%) was added to the aqueous phase and emulsified by a high-speed homogenizer (Ultra-Turrax T25, IKA-Werke, Staufen, Germany) at 19,000 rpm for 10 min in an ice bath to form the primary emulsion. The primary emulsion was further homogenized using a high-pressure homogenizer (EmulsiFlex-C3, Avestin Inc., Ottawa, ON, Canada) at 15,000 psi for 8 cycles to obtain the final emulsion for subsequent experiments.

Aliquots of 500 μL emulsion were dispensed into a 24-well plate and irradiated with UV light at a wavelength of 302 nm (5 mJ/cm^2^). After irradiation, the emulsion samples were mixed with ethanol and n-hexane at a volume ratio of 2:7:4, followed by centrifugation at 4000× *g* for 3 min. A 200 μL aliquot of the supernatant was transferred into a 96-well plate, and the absorbance was measured at 450 nm using a microplate reader to evaluate the photostability of β-carotene in the emulsion. The protection rate was assessed using the following formula:

Protection rate (%) = (As/Ac) × 100,

where Ac is the absorbance of the control and As is the absorbance of the sample.

### 3.18. Statistical Analysis

The data were statistically analyzed using analysis of variance (ANOVA), and mean differences were determined using Duncan’s multiple range test (*p* < 0.05) with SPSS Statistics 23 (IBM Corp., Armonk, NY, USA).

## 4. Conclusions

This study demonstrates the substantial potential of molecular modification of CT through conjugation with OLA and CAF to enhance its functional performance in oil-in-water emulsions. The grafting of OLA effectively increases the amphiphilicity, interfacial activity, and emulsifying efficiency of CT, enabling the formation of emulsions with improved stability, reduced droplet size, and enhanced resistance to environmental stresses such as ionic strength and heating. Complementarily, the incorporation of CAF introduces redox-active catechol groups and strong UVB-absorbing functionality, which further strengthens the oxidative and photo-stability of encapsulated lipophilic compounds such as β-carotene. The synergistic integration of hydrophobic modification (OLA) and antioxidant phenolic grafting (CAF) onto the CT backbone represents a powerful strategy for tailoring biopolymer-based emulsifiers with multifunctional attributes.

## Figures and Tables

**Table 1:** Effects of reactant ratios on the grafting efficiency of CT–OLA and the properties of the resulting conjugates.

Sample	CT (g) or CT-OLA (g)	OLA (mmol)	EDC (mmol)	NHS (mmol)	Precipitation ^1^	Amino- Substitution (%)	Yield (%)	Viscosity (cP)	Surface Tension (mN/m)
CT-OLA-1	1	2.6	2.6	2.6	++	-	-	-	-
CT-OLA-2	1	1.3	1.3	1.3	+	-	-	-	-
CT-OLA-3	1	1.3	2.6	2.6	++	-	-	-	-
CT-OLA-4	1	1.3	1.95	1.95	+++	-	-	-	-
CT-OLA-5	1	1.3	0.9625	0.9625	-	21.61 ± 0.36 ^c 2^	77.02 ± 1.21 ^a^	4.77 ± 0.08 ^c^	60.39 ± 0.17 ^c^
CT-OLA-6	1 (CT-OLA-5)	1.3	0.9625	0.9625	-	33.40 ± 0.50 ^b^	74.10 ± 1.07 ^b^	9.52 ± 0.16 ^b^	52.54 ± 0.27 ^d^
CT-OLA-7 ^2^	1 (CT-OLA-6)	1.3	0.9625	0.9625	-	46.74 ± 0.30 ^a^	71.74 ± 1.01 ^c^	13.17 ± 0.15 ^a^	50.32 ± 0.10 ^e^
CT								4.37 ± 0.02 ^d^	65.44 ± 0.17 ^b^
water									72.38 ± 0.34 ^a^

^1^ +++ > ++ > +: decreasing precipitation intensity; -: no precipitation. ^2^ Values (mean ± SD, n = 3) in the same column with different superscript letters are significantly different (*p* < 0.05).

**Table 2:** Effects of reactant ratios on grafting efficiency in the synthesis of CT–OLA–CAF and the properties of the resulting conjugates.

Sample ^1^	CT-OLA (g)	CAF (mmol) (g)	EDC (mmol) or Ascorbic Acid (g)	NHS (mmol) or H_2_O_2_ (M)	Grafting Ratio (%)	Viscosity (cP)	Surface Tension (mN/m)	Zeta Potential (mv)
E-CT-OLA-CAF-1	0.5	2.6	1.3	1.3	8.56 ± 0.05 ^a 2^	12.43 ± 0.25 ^b^	46.61 ± 0.30 ^f^	70.60 ± 0.17 ^a^
E-CT-OLA-CAF-2	0.5	2.6	2.6	2.6	6.34 ± 0.10 ^c^	12.83 ± 0.06 ^a^	48.32 ± 0.17 ^e^	70.07 ± 0.45 ^a^
E-CT-OLA-CAF-3	0.5	2.6	5.2	5.2	4.41 ± 0.03 ^e^	12.47 ± 0.25 ^b^	50.41 ± 0.15 ^d^	70.27 ± 0.49 ^a^
A-CT-OLA-CAF-1	0.5	0.25	0.054	0.5	6.89 ± 0.07 ^b^	1.02 ± 0.22 ^d^	35.82 ± 0.11 ^h^	57.57 ± 0.25 ^b^
A-CT-OLA-CAF-2	0.5	0.25	0054	1	4.81 ± 0.07 ^d^	1.06 ± 0.00 ^d^	37.61 ± 0.11 ^g^	57.33 ± 0.06 ^b^
CT-OLA						12.03 ± 0.29 ^b^	52.54 ± 0.27 ^c^	70.00 ± 0.50 ^a^
CT						5.11 ± 0.01 ^c^	65.44 ± 0.17 ^b^	57.67 ± 0.25 ^b^
water							72.38 ±0.34 ^a^	

^1^ E and A before CT-OLA-CAF denote preparation by amide coupling and free radical methods, respectively. ^2^ Values in the same column with different superscript letters are significantly different (*p* < 0.05).

**Table 3:** Antioxidant activities of CT-OLA-CAF determined by different assays.

Sample	Assays			
	DPPH (IC_50_: μg/mL)	H_2_O_2_ (IC_50_: μg/mL)	Fe^3+^ Reducing Power (Ascorbic Acid)	Fe^3+^ Reducing Power (Caffeic Acid)
E-CT-OLA-CAF-1	1001.71 ± 0.13 ^e 1^	1104.56 ± 2.20 ^e^	195.31 ± 0.64 ^a^	78.11 ± 0.55 ^a^
E-CT-OLA-CAF-2	1261.13 ± 0.90 ^c^	1193.02 ± 0.76 ^c^	158.82 ± 0.28 ^c^	64.06 ± 0.33 ^c^
E-CT-OLA-CAF-3	1747.50 ± 0.60 ^a^	1466.65 ± 0.43 ^a^	119.05 ± 0.82 ^e^	48.32 ± 0.59 ^e^
A-CT-OLA-CAF-1	1071.45 ± 0.35 ^d^	1164.79 ± 1.07 ^d^	188.29 ± 0.66 ^b^	75.73 ± 0.54 ^b^
A-CT-OLA-CAF-2	1393.60 ± 2.15 ^b^	1322.51 ± 2.45 ^b^	136.54 ± 0.87 ^d^	55.24 ± 0.60 ^d^
CT-OLA	>3000	>3000	- ^2^	-
CAF	151.94 ± 0.49 ^f^	48.89 ± 0.30 ^f^	-	-
CT	>3000	>3000	-	-

^1^ Values in the same column with different superscript letters are significantly different (*p* < 0.05). ^2^ Items are not analyzed.

**Table 4:** Oil-droplet size and distribution of O/W emulsions stabilized by CT and its conjugates before and after accelerated stability tests.

	Untreatment			Heat Treatment		Centrifugation	
Sample	Droplet Size (nm)	PDI	Viscosity (cP)	Droplet Size (nm)	PDI	Droplet Size (nm)	PDI
CT	881.17 ± 8.47 ^a 1^	0.24 ± 0.03	5.85 ± 0.02 ^A 2^	933.40 ± 5.10 ^c^	0.24 ± 0.01	913.10 ± 4.33 ^b^	0.25 ± 0.06
CT-OLA	482.80 ± 2.44 ^a^	0.13 ± 0.02	5.86 ± 0.02 ^A^	508.27 ± 2.20 ^b^	0.10 ± 0.06	482.60 ± 1.78 ^a^	0.12 ± 0.02
E-CT-OLA-CAF	463.60 ± 1.73 ^a^	0.14 ± 0.04	5.84 ± 0.02 ^A^	477.57 ± 10.00 ^b^	0.12 ± 0.04	464.90 ± 3.16 ^a^	0.13 ± 0.01
A-CT-OLA-CAF	211.17 ± 1.36 ^a^	0.06 ± 0.02	1.34 ± 0.01 ^B^	244.43 ± 0.45 ^b^	0.06 ± 0.03	211.43 ± 0.67 ^a^	0.08 ± 0.04

^1^: Values in the same row with different lowercase superscript letters are significantly different (*p* < 0.05). ^2^: Values in the same column with different uppercase superscript letters are significantly different (*p* < 0.05).

## Data Availability

The original contributions presented in this study are included in the article.
